# Morphological and Immunohistochemical Support for the Origin of the Carcinoid Component in Strumal Carcinoids: A Case Report and Literature Review

**DOI:** 10.3390/diagnostics15101249

**Published:** 2025-05-14

**Authors:** Yu Liu, Asra Feroze, Liz Yang, Ridin Balakrishnan

**Affiliations:** Department of Pathology, LSUHSC School of Medicine, New Orleans, LA 70112, USA; yliu6@lsuhsc.edu (Y.L.); aferoz@lsuhsc.edu (A.F.); lyang2@lsuhsc.edu (L.Y.)

**Keywords:** ovary, strumal carcinoid, strumal ovarii, thyroid, single cell pattern, TTF-1, PAX-8, CD56, synaptophysin

## Abstract

Strumal carcinoid tumors of the ovary are rare neoplasms composed of an intimate mixture of thyroid and carcinoid tissues. Although various theories regarding their histogenesis have been proposed, evidence confirming the origin of the carcinoid component has been lacking. We report a case of a 40-year-old female with an ovarian strumal carcinoid arising in the background of a mature cystic teratoma. Morphological and immunohistochemical findings support the hypothesis that the carcinoid component originates from the thyroid follicular epithelium, undergoing neuroendocrine differentiation. A single-cell growth pattern was also identified, expanding the known histological spectrum of strumal carcinoids. Our case provides additional immunohistochemical support for the histogenetic origin of strumal carcinoids, offering new insights into their pathogenesis. Recognizing these distinct patterns of staining and unusual morphology is critical for accurate diagnosis and differentiation from metastatic disease.

## 1. Introduction

Strumal carcinoid tumors of the ovary are exceedingly rare neoplasms that present a unique diagnostic and conceptual challenge. Histologically, these tumors are characterized by an intimate admixture of thyroid tissue (struma ovarii) and neuroendocrine (carcinoid) elements. There has been debate regarding whether strumal carcinoids are carcinoids with areas resembling thyroid tissue or whether they arise from bona fide thyroid tissue in the struma. Initial studies, including Robboy and Scully’s analysis of 50 cases [1], suggested a shared endodermal lineage based on the close intermixture of both components and their overlapping morphology. Subsequent investigations, including immunohistochemical and ultrastructural studies, proposed a unified origin, showing co-expression of thyroid markers (such as TTF-1 and thyroglobulin) and neuroendocrine markers (synaptophysin, chromogranin-A) in the carcinoid areas or transitional zones [2,3,4,5,6,7]. In contrast, others have proposed alternative theories, including their origin from parafollicular C-cells [8,9] or the notion that these tumors are pure neuroendocrine tumors but resemble a follicular architecture [10,11]. Despite these efforts, no definitive consensus has been reached.

Herein, we describe a case of a strumal carcinoid and use a panel of markers to show its morphological and immunohistochemical features, supporting the evolution of the neoplasm from struma ovarii to struma carcinoid within the same lesion. We also report a unique component of a single-cell growth pattern apart from the usual insular, trabecular, and mucinous patterns.

## 2. Case Presentation

The patient is a 40-year-old female with a history of chronic constipation who presented with abdominal and pelvic pain, fever, and episodes of hypotension. She reported a longstanding history of severe constipation, previously requiring hospitalization and manual disimpaction after a period of three weeks without bowel movement. She also experienced intermittent bright red blood per rectum, typically following hard bowel movements associated with straining. She denied unintentional weight loss and reported no family history of gastrointestinal malignancies.

**Figure 1:**
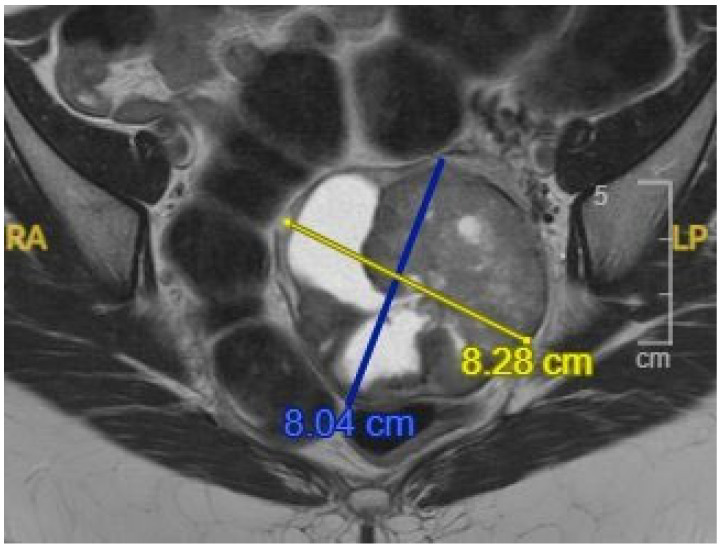
Abdominopelvic magnetic resonance imaging (MRI) shows an adnexal mass, with enhancement.

A computed tomography (CT) scan performed during her hospitalization incidentally identified a left ovarian mass with solid and cystic components and ovarian enlargement. Subsequent magnetic resonance imaging (MRI) revealed a well-marginated, mixed solid and cystic 8.3 cm left ovarian mass with enhancement and diffusion restriction in the solid components, raising suspicion for malignancy, including sex cord-stromal or germ cell tumors (Figure 1). Tumor markers (Table 1), including cancer antigen125 (CA125), carcinoembryonic antigen (CEA), carbohydrate antigen 19-9 (CA19-9), Inhibin B, and alpha-fetoprotein (AFP), were within normal limits, except for a mild elevation of lactate dehydrogenase (LDH). No evidence of metastatic disease was observed on contrast-enhanced MRI.

The patient was referred to gynecologic oncology and underwent a total laparoscopic hysterectomy, bilateral salpingo-oophorectomy, pelvic and para-aortic lymph node dissection, omentectomy, and staging biopsies.

Grossly, the left ovary weighed 155 g and measured 9.5 × 7.8 × 4.5 cm. Cross-sectioning revealed a round, yellow-tan, variegated, lobulated lesion with a focal cystic area measuring 2.5 × 2.0 × 1.5 cm, which was filled with yellow-tan mucoid fluid. Within this lesion, an additional well-circumscribed yellow-tan to brown-tan area was identified, containing necrotic cheesy material and hair. The right ovary exhibited a biloculated cyst measuring 3.4 × 2.8 × 1.4 cm, filled with yellow-tan fluid and lined with a smooth, unremarkable wall.

**Figure 2:**
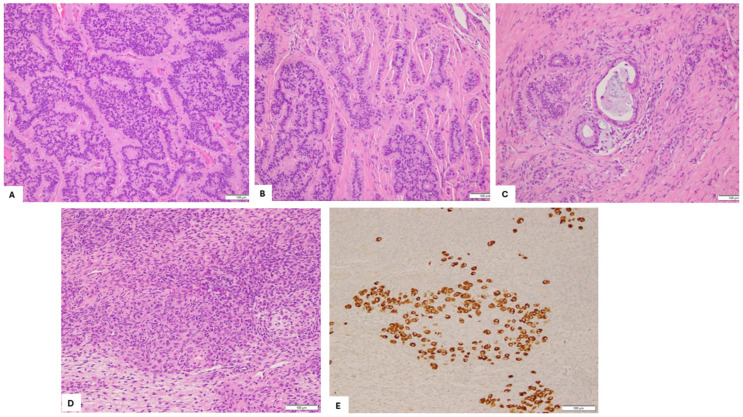
Histopathological patterns of the carcinoid tumor (HE, 200×). (**A**) Insular pattern displaying large nests and acini embedded within fibrous stroma; (**B**) Mixed trabecular and insular pattern with interweaving trabeculae and large nests of tumor cells; (**C**) Focal mucinous pattern with tumor cells demonstrating intracellular and extracellular mucin accumulation; (**D**) Isolated single-cell growth pattern observed at the ovarian surface, away from the primary tumor mass; (**E**) Immunohistochemistry staining positive for synaptophysin in single-cell growth pattern.

Microscopic analysis identified thyroid tissue consistent with struma ovarii, juxtaposed with a neuroendocrine neoplasm, all arising in the background of a mature cystic teratoma. The strumal component displayed no features of papillary or follicular thyroid carcinoma. The carcinoid component predominated and showed foci with large islands of variable-sized nests and acini within the fibrous stroma (Figure 2A insular pattern) and other areas that showed a mixed trabecular and insular pattern (Figure 2B). Focal areas with mucinous patterns (Figure 2C) were also identified. Additionally, an unusual single-cell growth pattern was identified at the ovarian surface, away from the main tumor (Figure 2D). Although these cells mildly resembled luteinized ovarian stromal cells, they stained positive for synaptophysin, as did the carcinoid tumor (Figure 2E).

**Figure 3:**
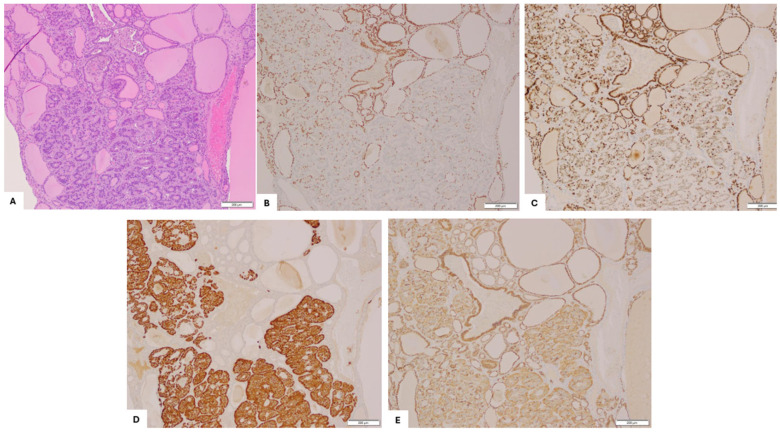
Strumal carcinoid arising from struma ovarii. (**A**) H&E, 100×; immunohistochemistry staining (100×). (**B**) Strong nuclear TTF-1 expression and (**C**) strong PAX8 expression in the strumal component, with gradual loss of TTF-1 and retained or weak PAX8 expression observed in the transition into the carcinoid component. (**D**) Synaptophysin is strongly positive in the carcinoid component, with few scattered positive cells in the strumal component. (**E**) CD56 shows membranous staining in the thyroid follicular epithelium of the struma and both basal and cytoplasmic expression in the carcinoid tumor cells.

Immunohistochemical staining (Figure 3), performed with adequate controls, showed that the struma was strongly positive for CK-7, TTF-1, PAX-8, and CD56 (membrane), and negative for synaptophysin, chromogranin-A, CDX-2, and GATA-3. In contrast, the carcinoid was focally interspersed positive for TTF-1, patchy positive for CDX-2 and PAX-8, and CD56 (basal and cytoplasmic), strongly positive for synaptophysin, and negative for GATA-3, calretinin, and chromogranin-A.

The interface between struma ovarii and carcinoid showed strong PAX-8 and uniform TTF-1 staining in the struma but gradual loss of TTF-1 (Figure 3B). Retained/weak PAX-8 staining (Figure 3C) was noted, moving away from the struma component and into the carcinoid. Synaptophysin was positive in the carcinoid component, with few scattered cells staining in the strumal component (Figure 3D). CD56 expression also demonstrated a phenotypic shift, showing strong membranous staining in the thyroid follicular epithelium but basal and cytoplasmic localization in the carcinoid component (Figure 3E).

All immunohistochemical staining was performed using standardized protocols with the following antibodies: CK-7 (clone OV-TL 12/30, Roche, 1:100), PAX-8 (clone MRQ-50, Cell Marque, 1:100), TTF-1 (clone 8G7G3/1, Roche, 1:100), CD56 (clone 123C3, Cell Marque, 1:50), synaptophysin (clone SP11, Roche, 1:50), chromogranin-A (clone LK2H10, Roche, 1:100), CDX-2 (clone EPR2764Y, Roche, 1:200), and GATA-3 (clone L50-823, Cell Marque, 1:100).

Given the achievement of complete surgical resection with negative margins, no adjuvant therapy was indicated, as the prognosis for strumal carcinoid tumors is generally favorable. The patient was closely monitored, and at 12 months follow-up, remained disease-free and reported normal bowel function without the need for medication.

## 3. Discussion and Literature Review

Strumal carcinoid tumors of the ovary are exceedingly rare germ cell neoplasms composed of an admixture of thyroid tissue and carcinoid tissues. Since the first case was described in 1970 [12], their carcinoid component origin has remained a topic of debate. Two main hypotheses have emerged: one proposing a shared origin from a single endodermal progenitor cell within struma ovarii, and another suggesting that the thyroid and carcinoid components represent distinct, coinciding neoplasms, possibly including metastasis from the gastrointestinal tract. For this review, we conducted a literature search using the keywords “ovarian strumal carcinoid” in PubMed and Google Scholar. We identified fifteen relevant publications (Table 2), including case reports, case series, and systematic reviews. Priority was given to studies utilizing immunohistochemistry or ultrastructural methods to investigate tumor histogenesis, as well as those offering indirect evidence through marker expression patterns.

The predominant theory in the literature supports a common origin from thyroid follicular epithelium. Robboy and Scully’s landmark analysis of 50 cases [1] first described argentaffin cells lining thyroid follicles containing birefringent colloid crystals, suggesting a histogenetic link. Additional ultrastructural studies found neuroendocrine granules in thyroglobulin-expressing follicular cells [2], while others identified transitional zones containing hybrid cells with both thyroid and neuroendocrine features [2,13]. Immunohistochemical co-expression of TTF-1, thyroglobulin, synaptophysin, and chromogranin in both tumor components, especially in these interface zones, further supports a unified lineage [2,7,14,15].

Our case aligns with this theory. The struma component showed strong CK-7, PAX-8, and TTF-1 positivity, confirming its thyroid origin. In contrast, the adjacent carcinoid component displayed classic insular and trabecular growth, with strong expression of synaptophysin and CD56. A gradual loss of TTF-1 and reduced PAX-8 expression was observed in the transition zone, alongside emerging neuroendocrine marker expression, providing morphologic and immunophenotypic evidence of thyroid-to-carcinoid transformation. The CD56 staining pattern, membranous in struma and basal/cytoplasmic in carcinoid areas, further supports a phenotypic shift during differentiation. Our morphological and immunohistochemical findings do not support a tumorigenic origin from a common endodermal progenitor cell. If such a progenitor were involved, a diffuse admixture of thyroid and carcinoid elements with intermediate or hybrid features would be expected. Instead, both components in our case were present in discrete, non-overlapping multiple foci. The carcinoid component appeared to arise focally within areas of struma ovarii, on the background of the thyroid follicular epithelium. This multifocal but spatially restricted pattern of neuroendocrine differentiation supports derivation from the thyroid epithelium rather than from a pluripotent endodermal precursor.

Alternative theories include derivation of the carcinoid component from parafollicular C-cells within struma ovarii, based on elevated serum calcitonin levels and immunohistochemical staining, reported in some cases [8,9,16]. However, this finding is inconsistent. Theurer et al. [7] and others failed to demonstrate calcitonin in either component [4,6,15,17,18]. Moreover, the absence of amyloid deposition or cytologic features typical of medullary thyroid carcinoma argues against a C-cell or medullary-type origin. Our findings further support this conclusion, as the carcinoid component showed retained but patchy expression of PAX8 and TTF-1 and a transitional zone with the strumal component, which are inconsistent with a parafollicular or neuroectodermal lineage origin and instead favor derivation from the thyroid follicular epithelium.

A less-supported hypothesis suggests that strumal carcinoids are purely neuroendocrine tumors mimicking thyroid architecture. Ultrastructural studies have shown neurosecretory granules in both components [10,11], but consistent immunostaining for thyroid markers such as TTF-1 and thyroglobulin in the strumal component refutes this idea and confirms genuine thyroid differentiation.

Our case also demonstrated several notable secondary findings. Patchy CDX2 expression, negative expression of chromogranin-A, and focal mucinous features in the carcinoid component suggest a gastrointestinal-type differentiation, consistent with prior reports [19,20]. The patient’s history of chronic severe constipation raises the possibility of peptide YY (PYY) secretion, which has been associated with strumal carcinoids in the literature [21,22,23], though this was not directly tested in our case. Additionally, we identified a previously undescribed single-cell growth pattern at the ovarian surface. These infiltrative, cytologically atypical, synaptophysin-positive cells must be distinguished from benign luteinized stromal cells and metastatic carcinoma. Awareness of this pattern is imperative, especially in the context of frozen sections, since an overcall can result in unnecessary staging in an otherwise mostly benign entity.

Recent meta-analysis data from 118 patients with ovarian strumal carcinoid indicate that, although the overall prognosis is favorable, metastatic disease and recurrence can occur [16]. Metastases were present at diagnosis in 3% of patients, and 4.5% experienced recurrence following radical surgery. While no clinicopathological factors were statistically linked to recurrence, patients who relapsed more often displayed larger tumors (median size 11 cm) and trabecular or mixed trabecular/insular carcinoid histology [16]. These observations suggest that long-term surveillance may be warranted, even in tumors with an otherwise indolent clinical course.

In summary, our case aligns with the growing body of literature suggesting that strumal carcinoids represent a distinct ovarian neoplasm originating from a single histogenetic pathway. The presence of a transitional zone, which retains thyroid-specific characteristics while progressively adopting neuroendocrine traits, supports the hypothesis that the carcinoid component arises directly from the thyroid follicular epithelium within struma ovarii. However, as molecular or genetic analysis was not performed, we are unable to provide definitive evidence of a clonal relationship between the strumal and carcinoid components, which represents a limitation of the study.

**Table 2:** Summary of studies on the origin of strumal carcinoid tumors of the ovary.

Reference	Number of Cases	Immunostaining/Ultrastructural Findings in Strumal Component	Immunostaining/Ultrastructural Findings in Carcinoid Component	Proposed Carcinoid Origin Hypothesis
S. Robbo [1]	50	-Calcium oxalate crystals present in half cases. Cells with argentaffin granules lined thyroid follicles. Positive for TG (n = 2/50).	Argentaffin granules and electron-dense granules are present.	Support unified origin.
Takubo, K. et al., 1986 [3]	1	Neurosecretory-type granules in the cytoplasm of thyroid follicular. Positive for TG.	Neurosecretory-type granules in the cytoplasm of carcinoid.	Support unified origin.
Tsubura, A., 1986 [4]	1	Positive for TG.	Carcinoid cells expanding toward the inner layer of the follicles. Negative for CAL and amyloid.	Support unified origin. Does not support origin from C-cells.
Stagno, P. et al., 1987 [5]	6	Positive for TG (6/6) Thyroid follicular epithelium lined with non-neuroendocrine cells.	Positive for CGA (5/6) and CAL (1/6). Carcinoid cells progressively replace thyroid epithelial cells.	Support origin from thyroid tissue in strumal ovarii.
S. Theurer et al., 2020 [7]	13	Positive for TG (100%), TTF-1(84.6%), CGA (76.9%), SYN (7.7%). Negative for CDX2, CAL SSTR2a, and SSTR5.	Positive for TG (100%), TTF-1(84.6%), CGA (92.3%), SYN (100%), SSTR2a (92.3%), SSTR5 (46.1%). Negative for CDX2 and CAL.	Support origin from thyroid tissue in strumal ovarii. Do not support origin from C-cells.
L. Greco et al. 1979 [14]	1	Positive for TG in thyroid follicles and transitional zone.	Negative for TG. Positive for CAL.	Support origin from thyroid tissue in strumal ovarii.
M. Senterman et al., 1984 [15]	1	Positive for TG in thyroid follicles and transitional zone. Negative for CAL.	Negative for TG and CAL.	Support origin from thyroid tissue in strumal ovarii. Do not support origin from C-cells.
R. Snyder et al., 1986 [2]	13	Positive for TG in thyroid follicles and transitional zone (12/12). NE granules in thyroid follicles (6/6).	Negative for TG and CA (12/12). NE granules in carcinoid (6/6).	Support origin from thyroid tissue in strumal ovarii. Do not support origin from C-cells.
Kimura, N. et al., 1986 [13]	1	Strong positive for TG. Intensive argyrophilia. Abundant neurosecretory granules, microfilaments, colloid-like droplets in thyroid follicles.	Weak positive for TG, intensive argyrophilia. Abundant neurosecretory granules, microfilaments, colloid-like droplets in carcinoid.	Support origin from hybrid cells having both thyroid and neuroendocrine features.
S. Hamazaki et al., 2002 [6]	2	Positive for TTF-1, TG. Negative for CGA, SYN, PAP, CAL.	-Positive for CGA, SYN, PAP. Negative for TTF-1, TG, CAL.	Divergent differentiation from a common progenitor. Do not support origin from C-cells.
K. Macháleková, 2018 [18]	2	Positive for TTF-1, TG. Negative for CAL, CEA.	Positive for CGA, SYN. Negative for CAL, CEA.	Do not support origin from C-cells.
C. Apostol et al., 2017 [17]	1	Positive for TTF-1, TG, CD56. CK7, CK19. Negative for CAL, HBME1, GFAP.	Positive for SYN, CGA, CD56. Negative for CK7, CAL, GFAP.	Do not support origin from C-cells.
Blaustein, A., 1979 [8]	1	N/A	Positive for CAL and 5-HA. Ultrastructural similarity to medullary carcinoma.	Support origin from C- cells.
Ulbright, T. et al., 1982 [9]	2	Granule-containing cells replaced some follicular epithelial cells.	Positive for CAL in transitional zone and carcinoid.	Support origin from C-cells.
A.Turla et al., 2022 [16]	117	Positive for TG (41/45), TTF-1(22/25).	Positive for CGA (39/39), SYN (31/31), CD56 (16/16), NSE (13/13), CAL (12/23).	Support origin from C-cells.

TG: Thyroglobulin; TTF-1: thyroid transcription factor-1; CGA: chromogranin A; SYN: synaptophysin; CAL: calcitonin; SSTR2a: somatostatin receptor 2a; SSTR5: somatostatin receptor 5; CEA: carcinoembryonic antigen; CK7: cytokeratin 7; CK19: cytokeratin 19; CD56: neural cell adhesion molecule; NSE: neuron-specific enolase; 5-HA: 5-hydroxytryptamine (serotonin).

## 4. Conclusions

We provide morphological and immunohistochemical evidence that strumal carcinoids likely develop from thyroid tissue undergoing neuroendocrine differentiation in struma ovarii. Specifically, retained/weak PAX-8 with interspersed TTF-1 in an adnexal carcinoid is a distinct pattern to be aware of when dealing with carcinoid tumors of unknown etiology, strongly favoring an ovarian primary origination (specifically, from struma ovarii) and not metastasis from the gastrointestinal tract or other Mullerian tissues. Recognizing this potential origin is crucial for the diagnosis, management, and understanding of the tumor’s behavior. Lastly, the single-cell growth pattern we have identified broadens the known morphological spectrum of strumal carcinoid tumors and is a significant feature to consider, especially in the context of metastases or intraoperative frozen sections.

## Figures and Tables

**Table 1:** Preoperative tumor marker levels for the patient.

Tumor Marker	Results	Reference Range
CA125	12.3 U/mL	<35 U/mL
CEA	1.3 ng/mL	<5 ng/mL
CA19-9	7.7 U/mL	<35.1 U/mL
Inhibin B	106 pg/mL	<261 pg/mL
AFP	2.1 ng/mL	<15 ng/mL
LDH	243 U/L	<201 U/L

## Data Availability

The original contributions presented in this study are included in the article. Further inquiries can be directed to the corresponding author.
